# Molecular characterization and phylogenetic analysis of bovine ephemeral fever viruses in Khuzestan province of Iran in 2018 and 2020

**DOI:** 10.1186/s12917-021-03119-x

**Published:** 2022-01-06

**Authors:** Seyedeh Elham Rezatofighi, Khalil Mirzadeh, Fahimeh Mahmoodi

**Affiliations:** 1 Biology department, Faculty of Science, Shahid Chamran University of Ahvaz, Ahvaz, 6135743135 Iran; 2 Department of Animal Science, Faculty of Animal Sciences and Food Technology, Agricultural Sciences and Natural Resources University of Khuzestan, Mollasani, Iran

**Keywords:** Bovine ephemeral fever virus, Phylogenetic analysis, Molecular characterization, G gene, Iran, Middle East

## Abstract

**Background:**

Bovine ephemeral fever (BEF) is an arthropod-borne viral disease caused by the BEF virus (BEFV). This single-stranded RNA virus that affects cattle and water buffalo is endemic in tropical and subtropical regions including Iran. While BEF is a major disease of cattle in Iran, information regarding its agent, molecular characterization, and circulating viruses are highly limited. The current study aimed to, firstly, determine the genetic and antigenic characteristics of BEFV strains in Khuzestan province in Southwest of Iran in 2018 and 2020 and, secondly, to compare them with strains obtained from other areas.

**Results:**

By phylogenetic analysis based on the Glycoprotein gene, BEFV strains were divided into four clusters of Middle East, East Asia, South Africa, and Australia; in which the 2018 and 2020 Iranian BEFV strains were grouped in the Middle East cluster with the Turkish, Indian, and Israeli strains. Depending on the chronology and geographical area, the outbreaks of Turkey (2020), Iran (2018 and 2020), and India (2018 and 2019) are proposed to be related. These BEFVs had the highest identity matrix and the lowest evolutionary distance among the studied strains. Multiple sequence alignment of G1, G2, and G3 antigenic sites showed that these neutralizing epitopes are highly conserved among the strains of the Middle East cluster; however, the strains previously identified in Iran differed in three amino acids placed in G1 and G2 epitopes.

**Conclusion:**

The findings revealed that BEFVs circulating in the Middle East are closely related phylogenetically and geographically. They also have similar antigenic structures; therefore, developing a vaccine based on these strains can be effective for controlling BEF in the Middle East.

**Supplementary Information:**

The online version contains supplementary material available at 10.1186/s12917-021-03119-x.

## Introduction

Bovine ephemeral fever (BEF), also called three-day sickness, is a viral disease that affects cattle and water buffalo. BEF virus (BEFV) is endemic in tropical and subtropical regions of Australia, Africa, and Asia, especially the Middle East countries [1, 2]. The disease is marked by a bi-phasic or multiple-phasic fever, anorexia, muscle stiffness, salivation, depression, lameness, ocular and nasal discharge, cessation of rumination, and constipation [3]. BEF causes considerable economic losses, mainly because of its consequences and imposing international trade restrictions [4, 5]. BEF, caused by an arthropod-borne virus, usually occurs in the summer and early autumn months [3]. Weather conditions plays a unique role in the spread of the BEF. Rainfall, prevailing wind patterns, streams, or other ground water sources have a significant effect on its occurrence, particularly in wet and dry seasons [6].

BEFV belongs to the genus *Ephemerovirus*, *Rhabdoviridae* family and has a negative-sense single-stranded RNA (ssRNA) [7]. The length of the viral genome is 14,900 nucleotides and encodes five structural proteins of the matrix, nucleoprotein, phosphoprotein, surface glycoprotein, and RNA-dependent RNA polymerase. Surface glycoprotein (G) is a class I transmembrane glycoprotein that includes the major neutralizing epitopes and can induce protective immunity in cattle [8, 9]. Four distinct antigenic sites (G1-G4) have been determined on the surface of this protein [9–11]. The viral capsid binds to the cell surface via G protein and penetrates into the cells through receptor-mediated endocytosis. Therefore, G protein plays an important role in the virus replication process as well as induction of immune response in the host body [6].

BEF disease is prevalent in Middle East countries and is considered an enzootic disease in some areas of this region [4, 12]. The first detailed report of BEF in the Middle East was described in Egypt in 1909, in which the disease spread from Aswan, Nile Valley to Cairo, and spread across the Delta to the coast [11]. Afterward, the disease was reported in the Palestine and Jordan Valley in 1931 [12, 13], followed by Israel, Turkey, Iraq, Saudi Arabia, Kuwait, Yemen, Syria, and Iran [12, 14–16]. In Iran, the first report of BEFV isolation date back to the occurrence of the virus in the southern and eastern regions of the country in 1974 [14]. The disease is mostly sporadic and is prevalent in southern and western areas with tropical climates [14]. Three outbreaks are also recorded in 2006 [17] and in autumn of 2012 and 2013 [14] in Iran, before the last outbreak seen in 2020. The disease was appeared in Khuzestan in March as sporadic cases and then gradually appeared in the other provinces and soon reached to at least fourteen provinces (unpublished data). The peak of the disease was observed in July and August. While no precise estimate is available about losses caused by the outbreak; 70% of livestock in the infected areas showed clinical signs of disease (unpublished data).

Although BEF is known to occur as a single serotype worldwide, some antigenic variations are detected at virus epitope sites [18]. Detection of these variations would be useful for identifying the epidemiology of BEFV, genetic evolution, and design broad-spectrum vaccines [18]. To determine the genetic and antigenic variations of BEFV strains in Iran in 2018 and 2020, analyzed the nucleotide and deduced amino acid sequences of complete G gene of BEFV isolates and compared the sequences with those of the GenBank database.

## Results

### Detection of BEFV

RT-nested PCR showed that all suspicious samples were positive for BEFV and a specific product of 420 bp was found. To determine the genetic characteristics of BEFVs circulating in Iran during 2018 and 2020, the glycoprotein encoding gene of 8 BEFV strains (4 strains related to 2018 and 4 strains related to 2020) were sequenced. Because the G gene sequences of the prevalent strains in each year were the same, only the sequence of one strain in each year was submitted to the GenBank. The accession numbers of MZ51169 and MZ51168 were acquired for 2018 and 2020 Iranian BEFV strains, respectively.

### Identity and evolutionary distance analysis

G gene nucleotide and deduced amino acid sequences of Iranian BEFV strains were compared with other BEFV sequences in the GenBank using multiple sequence alignment. The IR-2018 and IR-2020 strains showed the lowest identity with South African strains (86.8–87.1% nucleotide identity and 93–93.5% amino-acid identity). The IR-2018 strain shared the most nucleotide identity with the IR-2020 strain (98.4%), and Indian strains (98.2%), respectively, while concerning amino acid sequence, it showed the highest identity (99%) with the 2020 Turkish, 2006 Israeli and IR-2020 strains. The G gene sequence of the IR-2020 strain showed the highest nucleotide identity with strains identified in Turkey in 2020 (99.4–99.6% identity) as well as Indian strains (99.1% identity). Two BEFV strains of 2020 Iran and Turkey were quite similar in terms of amino acid sequence and were more than 99% similar to the Indian strains. Two investigated Iranian strains had 98.4 and 99% nucleotide and amino acid sequence identity, respectively (Table 1 and Supplementary Table 1).

**Table 1:** Distance estimation (left, bottom) and Identity Matrix (right, top) of BEFVs from different countries according to G gene

Seq->	1	2	3	4	5	6	7	8	9	10	11	12	13	14	15	16	17	18	19	20	21	22	23
1.MW387421. Turkey. 2020	ID	0.998	0.992	0.992	0.97	0.967	0.971	0.967	0.966	0.97	0.97	0.97	0.96	0.946	0.937	0.934	0.933	0.933	0.93	0.929	0.928	0.928	0.928
2.MW387420. Turkey. 2020	0.001	ID	0.991	0.991	0.969	0.966	0.969	0.965	0.965	0.969	0.969	0.969	0.958	0.946	0.937	0.934	0.933	0.933	0.93	0.929	0.928	0.928	0.928
3.MN839987. India. 2018	0.007	0.009	ID	1	0.973	0.97	0.971	0.969	0.969	0.97	0.97	0.97	0.962	0.943	0.935	0.931	0.931	0.93	0.927	0.926	0.925	0.925	0.925
4.MN905763. India. 2019	0.007	0.009	0.000	ID	0.973	0.97	0.971	0.969	0.969	0.97	0.97	0.97	0.962	0.943	0.935	0.931	0.931	0.93	0.927	0.926	0.925	0.925	0.925
5.MN078236. Israel. 2006	0.030	0.032	0.028	0.028	ID	0.996	0.973	0.995	0.994	0.972	0.972	0.972	0.984	0.952	0.941	0.939	0.939	0.939	0.935	0.935	0.934	0.934	0.934
6.JN833631. Israel. 2001	0.030	0.032	0.028	0.028	0.001	ID	0.972	0.998	0.997	0.971	0.971	0.971	0.986	0.952	0.941	0.938	0.939	0.938	0.934	0.934	0.933	0.933	0.933
7.GQ229452. Turkey. 2008	0.028	0.030	0.029	0.029	0.027	0.026	ID	0.971	0.971	0.996	0.996	0.999	0.965	0.95	0.94	0.939	0.939	0.938	0.936	0.932	0.935	0.931	0.935
8.JN833632. Israel. 2004	0.031	0.033	0.028	0.028	0.002	0.002	0.027	ID	0.996	0.971	0.971	0.971	0.986	0.951	0.94	0.937	0.938	0.937	0.933	0.933	0.933	0.933	0.933
9.JN833630. Israel. 2000	0.030	0.032	0.028	0.028	0.001	0.001	0.026	0.002	ID	0.97	0.97	0.97	0.985	0.95	0.939	0.937	0.937	0.937	0.933	0.933	0.932	0.932	0.932
10.KY012742. Turkey. 2012	0.029	0.031	0.030	0.030	0.028	0.027	0.004	0.028	0.027	ID	1	0.995	0.965	0.948	0.939	0.937	0.937	0.937	0.933	0.931	0.933	0.93	0.933
11.KC788421. Turkey. 2012	0.029	0.031	0.030	0.030	0.028	0.027	0.004	0.028	0.027	0.000	ID	0.995	0.965	0.948	0.939	0.937	0.937	0.937	0.933	0.931	0.933	0.93	0.933
12.GQ229451. Turkey. 2008	0.030	0.031	0.030	0.030	0.028	0.027	0.001	0.028	0.027	0.005	0.005	ID	0.964	0.949	0.939	0.939	0.938	0.937	0.935	0.931	0.935	0.931	0.935
13.JN833633. Israel. 2010	0.038	0.040	0.035	0.035	0.013	0.013	0.033	0.013	0.013	0.033	0.033	0.034	ID	0.948	0.935	0.934	0.935	0.933	0.929	0.93	0.929	0.929	0.929
14.KJ729108. Egypt. 2012	0.056	0.056	0.059	0.059	0.048	0.048	0.052	0.049	0.048	0.053	0.053	0.053	0.051	ID	0.986	0.968	0.967	0.965	0.963	0.964	0.965	0.963	0.967
15.AB462028. Japan. 1966	0.065	0.065	0.068	0.068	0.061	0.060	0.063	0.061	0.060	0.065	0.065	0.064	0.065	0.014	ID	0.975	0.974	0.972	0.97	0.97	0.973	0.969	0.973
16.AB462036. Japan. 1988	0.069	0.069	0.072	0.072	0.064	0.063	0.064	0.064	0.063	0.066	0.066	0.065	0.067	0.032	0.025	ID	0.998	0.996	0.994	0.979	0.98	0.979	0.982
17.AB462039. Japan. 1989	0.070	0.070	0.073	0.073	0.063	0.062	0.065	0.063	0.063	0.067	0.067	0.065	0.066	0.033	0.026	0.002	ID	0.995	0.993	0.98	0.98	0.979	0.981
18.AB462037. Japan. 1989	0.071	0.071	0.074	0.074	0.064	0.063	0.065	0.064	0.063	0.066	0.066	0.066	0.068	0.035	0.028	0.004	0.005	ID	0.991	0.977	0.978	0.976	0.979
19.KJ605423. Taiwan. 1984	0.074	0.074	0.077	0.077	0.068	0.068	0.068	0.069	0.068	0.072	0.072	0.069	0.072	0.037	0.030	0.006	0.007	0.009	ID	0.975	0.976	0.974	0.977
20.AY935241. Taiwan.2001	0.075	0.075	0.078	0.078	0.068	0.068	0.072	0.068	0.068	0.074	0.074	0.073	0.071	0.037	0.030	0.021	0.020	0.023	0.026	ID	0.99	0.999	0.991
21.KJ605424. Taiwan. 1996	0.076	0.076	0.079	0.079	0.069	0.068	0.068	0.069	0.068	0.071	0.071	0.069	0.072	0.035	0.027	0.019	0.020	0.022	0.024	0.010	ID	0.989	0.997
22.AB462043. Japan. 2001	0.076	0.076	0.079	0.079	0.069	0.068	0.073	0.069	0.068	0.074	0.074	0.074	0.072	0.037	0.031	0.021	0.021	0.024	0.026	0.001	0.011	ID	0.99
23.AY935240. Taiwan. 1996	0.076	0.076	0.079	0.079	0.069	0.068	0.068	0.069	0.068	0.071	0.071	0.069	0.072	0.034	0.027	0.018	0.019	0.021	0.023	0.009	0.003	0.009	ID
24.KP403933. Taiwan. 2012	0.077	0.077	0.080	0.080	0.071	0.071	0.074	0.071	0.071	0.075	0.075	0.074	0.075	0.042	0.039	0.029	0.028	0.032	0.034	0.009	0.018	0.010	0.017
25.KC470312. Turkey. 2012	0.081	0.081	0.084	0.084	0.076	0.075	0.076	0.076	0.075	0.078	0.078	0.077	0.079	0.044	0.037	0.028	0.029	0.030	0.032	0.025	0.022	0.026	0.019
26.MH105245. Thailand. 2016	0.084	0.084	0.087	0.087	0.080	0.080	0.081	0.080	0.080	0.083	0.083	0.082	0.082	0.052	0.044	0.035	0.035	0.038	0.040	0.019	0.027	0.020	0.025
27.KP403937. Taiwan. 2013	0.084	0.084	0.084	0.084	0.077	0.077	0.080	0.078	0.077	0.080	0.080	0.080	0.078	0.050	0.043	0.034	0.035	0.035	0.039	0.032	0.031	0.032	0.030
28.MH105230. Thailand. 2015	0.085	0.085	0.088	0.088	0.081	0.080	0.082	0.081	0.081	0.083	0.083	0.083	0.083	0.053	0.044	0.036	0.035	0.039	0.041	0.019	0.027	0.020	0.025
29.MF491476. Iran. 2013	0.086	0.086	0.087	0.087	0.080	0.080	0.083	0.080	0.080	0.083	0.083	0.083	0.082	0.053	0.046	0.035	0.036	0.035	0.040	0.033	0.034	0.032	0.032
30.MH756623. China. 2017	0.087	0.087	0.090	0.090	0.083	0.083	0.084	0.084	0.083	0.086	0.086	0.085	0.085	0.057	0.048	0.039	0.039	0.042	0.044	0.023	0.030	0.023	0.029
31.MF491475. Iran. 2012	0.087	0.087	0.089	0.089	0.082	0.081	0.083	0.082	0.081	0.082	0.082	0.084	0.084	0.053	0.046	0.037	0.037	0.037	0.041	0.035	0.034	0.035	0.032
32.KF679427. Australia. 1989	0.099	0.099	0.099	0.099	0.095	0.095	0.092	0.096	0.095	0.095	0.095	0.093	0.096	0.088	0.087	0.088	0.088	0.091	0.092	0.098	0.098	0.098	0.096
33.KF679437. Australia. 1956	0.098	0.098	0.099	0.099	0.094	0.094	0.092	0.095	0.094	0.095	0.095	0.093	0.095	0.083	0.080	0.084	0.084	0.087	0.089	0.092	0.089	0.093	0.087
34.KF679496. Australia. 1989	0.099	0.099	0.099	0.099	0.094	0.094	0.091	0.094	0.094	0.094	0.094	0.092	0.095	0.088	0.087	0.088	0.088	0.091	0.092	0.098	0.098	0.098	0.096
35.KF679494. Australia. 1989	0.100	0.100	0.100	0.100	0.096	0.096	0.093	0.097	0.096	0.096	0.096	0.094	0.097	0.088	0.087	0.088	0.089	0.091	0.093	0.098	0.098	0.099	0.097
36.KF679423. Australia. 1970	0.100	0.100	0.101	0.101	0.095	0.095	0.096	0.096	0.095	0.099	0.099	0.097	0.096	0.086	0.084	0.085	0.086	0.088	0.090	0.094	0.094	0.095	0.092
37.AF058325. Australia. 1970	0.101	0.101	0.102	0.102	0.096	0.096	0.097	0.097	0.096	0.100	0.100	0.098	0.097	0.087	0.085	0.086	0.085	0.089	0.090	0.095	0.095	0.095	0.093
38.KF679413. Australia. 1970	0.101	0.101	0.102	0.102	0.095	0.095	0.097	0.095	0.095	0.100	0.100	0.098	0.096	0.087	0.085	0.086	0.087	0.089	0.091	0.095	0.095	0.096	0.093
39.KF679468. Australia. 2010	0.110	0.110	0.109	0.109	0.099	0.099	0.102	0.098	0.099	0.103	0.103	0.103	0.102	0.096	0.096	0.095	0.096	0.097	0.100	0.105	0.106	0.105	0.104
40.KF679453. Australia. 2020	0.111	0.111	0.110	0.110	0.100	0.100	0.103	0.099	0.100	0.104	0.104	0.104	0.103	0.095	0.096	0.095	0.096	0.097	0.100	0.105	0.105	0.105	0.104
41.KF679456. Australia. 2010	0.112	0.112	0.111	0.111	0.099	0.099	0.102	0.099	0.099	0.104	0.104	0.103	0.103	0.096	0.097	0.096	0.097	0.096	0.100	0.105	0.106	0.106	0.105
42.MN026885. South Africa. 1969	0.137	0.139	0.139	0.139	0.134	0.134	0.145	0.135	0.134	0.145	0.145	0.146	0.140	0.135	0.133	0.132	0.133	0.134	0.138	0.135	0.139	0.136	0.139
43.MN026897. South Africa. 2018	0.139	0.141	0.140	0.140	0.134	0.134	0.145	0.135	0.133	0.145	0.145	0.146	0.140	0.135	0.132	0.132	0.133	0.135	0.139	0.138	0.142	0.139	0.141
44.MN026881. South Africa. 1969	0.139	0.141	0.140	0.140	0.136	0.136	0.147	0.137	0.135	0.147	0.147	0.148	0.142	0.136	0.134	0.133	0.134	0.135	0.139	0.136	0.140	0.137	0.139
45.MN026880. South Africa. 1968	0.140	0.142	0.142	0.142	0.138	0.138	0.147	0.138	0.137	0.147	0.147	0.148	0.144	0.138	0.136	0.136	0.137	0.138	0.142	0.140	0.144	0.140	0.143
46.MZ511168. Iran. 2020	0.003	0.005	0.008	0.008	0.030	0.030	0.031	0.030	0.030	0.032	0.032	0.032	0.037	0.057	0.067	0.071	0.072	0.072	0.076	0.077	0.078	0.078	0.078
47.MZ511169. Iran. 2018	0.017	0.019	0.015	0.015	0.027	0.026	0.029	0.027	0.026	0.030	0.030	0.030	0.032	0.060	0.070	0.074	0.075	0.074	0.079	0.079	0.080	0.080	0.080

Seq->	24	25	26	27	28	29	30	31	32	33	34	35	36	37	38	39	40	41	42	43	44	45	46	47
1.MW387421. Turkey. 2020	0.927	0.924	0.921	0.921	0.92	0.92	0.918	0.918	0.907	0.908	0.906	0.905	0.906	0.905	0.903	0.897	0.897	0.896	0.874	0.873	0.873	0.872	0.996	0.981
2.MW387420. Turkey. 2020	0.927	0.924	0.921	0.921	0.92	0.92	0.918	0.918	0.907	0.908	0.906	0.905	0.906	0.905	0.903	0.897	0.897	0.896	0.873	0.872	0.872	0.87	0.994	0.979
3.MN839987. India. 2018	0.925	0.921	0.918	0.921	0.918	0.918	0.916	0.917	0.906	0.907	0.905	0.905	0.905	0.905	0.903	0.898	0.897	0.897	0.873	0.872	0.872	0.87	0.991	0.982
4.MN905763. India. 2019	0.925	0.921	0.918	0.921	0.918	0.918	0.916	0.917	0.906	0.907	0.905	0.905	0.905	0.905	0.903	0.898	0.897	0.897	0.873	0.872	0.872	0.87	0.991	0.982
5.MN078236. Israel. 2006	0.932	0.928	0.924	0.927	0.924	0.924	0.922	0.923	0.909	0.91	0.91	0.908	0.91	0.909	0.908	0.906	0.905	0.906	0.876	0.876	0.875	0.874	0.97	0.971
6.JN833631. Israel. 2001	0.931	0.927	0.923	0.926	0.923	0.923	0.921	0.922	0.912	0.913	0.912	0.91	0.912	0.912	0.911	0.908	0.908	0.908	0.879	0.879	0.877	0.876	0.967	0.969
7.GQ229452. Turkey. 2008	0.931	0.928	0.924	0.925	0.923	0.923	0.922	0.922	0.912	0.913	0.913	0.911	0.91	0.909	0.907	0.904	0.904	0.904	0.868	0.868	0.867	0.867	0.968	0.969
8.JN833632. Israel. 2004	0.931	0.927	0.923	0.925	0.922	0.923	0.92	0.922	0.911	0.912	0.912	0.91	0.912	0.911	0.91	0.909	0.908	0.909	0.878	0.878	0.877	0.875	0.967	0.969
9.JN833630. Israel. 2000	0.93	0.926	0.922	0.925	0.922	0.922	0.92	0.921	0.91	0.912	0.911	0.909	0.911	0.91	0.91	0.907	0.906	0.907	0.878	0.878	0.877	0.875	0.966	0.968
10.KY012742. Turkey. 2012	0.929	0.927	0.923	0.925	0.922	0.923	0.92	0.923	0.91	0.91	0.91	0.908	0.907	0.906	0.904	0.903	0.903	0.903	0.868	0.868	0.867	0.867	0.967	0.968
11.KC788421. Turkey. 2012	0.929	0.927	0.923	0.925	0.922	0.923	0.92	0.923	0.91	0.91	0.91	0.908	0.907	0.906	0.904	0.903	0.903	0.903	0.868	0.868	0.867	0.867	0.967	0.968
12.GQ229451. Turkey. 2008	0.93	0.927	0.923	0.925	0.923	0.922	0.921	0.922	0.912	0.912	0.912	0.91	0.909	0.908	0.906	0.904	0.903	0.904	0.868	0.868	0.866	0.866	0.967	0.968
13.JN833633. Israel. 2010	0.927	0.923	0.921	0.925	0.92	0.921	0.918	0.92	0.91	0.912	0.911	0.909	0.911	0.91	0.91	0.906	0.905	0.904	0.875	0.875	0.873	0.872	0.96	0.963
14.KJ729108. Egypt. 2012	0.959	0.958	0.95	0.952	0.949	0.949	0.946	0.949	0.916	0.921	0.916	0.915	0.918	0.918	0.916	0.909	0.91	0.909	0.877	0.877	0.876	0.874	0.944	0.94
15.AB462028. Japan. 1966	0.962	0.964	0.958	0.958	0.957	0.956	0.954	0.956	0.917	0.923	0.916	0.916	0.92	0.919	0.917	0.909	0.909	0.908	0.878	0.879	0.878	0.876	0.935	0.931
16.AB462036. Japan. 1988	0.971	0.972	0.965	0.967	0.965	0.965	0.961	0.964	0.916	0.92	0.916	0.915	0.919	0.918	0.916	0.91	0.91	0.909	0.88	0.879	0.879	0.876	0.932	0.928
17.AB462039. Japan. 1989	0.972	0.971	0.966	0.966	0.965	0.965	0.962	0.963	0.916	0.92	0.915	0.914	0.918	0.919	0.916	0.909	0.909	0.908	0.879	0.878	0.878	0.876	0.931	0.927
18.AB462037. Japan. 1989	0.969	0.971	0.963	0.965	0.962	0.965	0.959	0.964	0.914	0.918	0.913	0.912	0.916	0.916	0.914	0.908	0.908	0.909	0.878	0.877	0.877	0.874	0.931	0.928
19.KJ605423. Taiwan. 1984	0.967	0.968	0.961	0.962	0.96	0.961	0.957	0.96	0.913	0.916	0.912	0.912	0.916	0.915	0.913	0.906	0.906	0.906	0.875	0.874	0.874	0.872	0.928	0.924
20.AY935241. Taiwan.2001	0.99	0.975	0.98	0.969	0.98	0.967	0.977	0.966	0.908	0.913	0.907	0.906	0.912	0.911	0.909	0.902	0.902	0.901	0.877	0.875	0.876	0.874	0.927	0.924
21.KJ605424. Taiwan. 1996	0.982	0.978	0.973	0.969	0.973	0.967	0.97	0.967	0.908	0.916	0.907	0.906	0.912	0.911	0.909	0.901	0.901	0.901	0.874	0.872	0.873	0.87	0.926	0.923
22.AB462043. Japan. 2001	0.99	0.974	0.98	0.968	0.98	0.968	0.977	0.965	0.907	0.912	0.906	0.906	0.911	0.91	0.908	0.901	0.901	0.901	0.876	0.874	0.876	0.873	0.926	0.923
23.AY935240. Taiwan. 1996	0.983	0.98	0.975	0.971	0.975	0.968	0.971	0.968	0.909	0.917	0.908	0.908	0.913	0.912	0.91	0.903	0.903	0.902	0.874	0.872	0.874	0.871	0.926	0.923
24.KP403933. Taiwan. 2012	ID	0.968	0.975	0.965	0.975	0.962	0.971	0.961	0.903	0.908	0.902	0.901	0.906	0.906	0.904	0.897	0.897	0.896	0.874	0.872	0.873	0.87	0.925	0.923
25.KC470312. Turkey. 2012	0.032	ID	0.961	0.978	0.961	0.987	0.958	0.987	0.905	0.913	0.904	0.904	0.908	0.907	0.905	0.899	0.899	0.899	0.87	0.87	0.87	0.868	0.922	0.918
26.MH105245. Thailand. 2016	0.025	0.040	ID	0.954	0.998	0.954	0.995	0.954	0.904	0.914	0.904	0.903	0.911	0.91	0.908	0.899	0.899	0.899	0.873	0.871	0.872	0.87	0.919	0.918
27.KP403937. Taiwan. 2013	0.036	0.022	0.047	ID	0.954	0.984	0.951	0.984	0.903	0.909	0.902	0.901	0.906	0.906	0.904	0.897	0.897	0.896	0.868	0.868	0.868	0.866	0.919	0.917
28.MH105230. Thailand. 2015	0.025	0.040	0.001	0.047	ID	0.954	0.995	0.954	0.904	0.913	0.903	0.903	0.91	0.91	0.908	0.899	0.899	0.898	0.874	0.872	0.873	0.87	0.918	0.918
29.MF491476. Iran. 2013	0.039	0.013	0.048	0.016	0.048	ID	0.95	0.996	0.901	0.908	0.901	0.9	0.904	0.903	0.901	0.895	0.895	0.895	0.87	0.869	0.869	0.867	0.918	0.916
30.MH756623. China. 2017	0.029	0.044	0.005	0.051	0.005	0.052	ID	0.95	0.902	0.911	0.901	0.901	0.908	0.908	0.906	0.897	0.897	0.896	0.872	0.87	0.871	0.868	0.916	0.916
31.MF491475. Iran. 2012	0.039	0.013	0.048	0.016	0.048	0.004	0.052	ID	0.901	0.909	0.901	0.9	0.904	0.903	0.901	0.895	0.895	0.895	0.868	0.868	0.868	0.866	0.916	0.916
32.KF679427. Australia. 1989	0.104	0.101	0.102	0.104	0.103	0.106	0.105	0.106	ID	0.966	0.998	0.997	0.986	0.984	0.982	0.979	0.978	0.978	0.878	0.879	0.877	0.879	0.907	0.908
33.KF679437. Australia. 1956	0.098	0.092	0.092	0.097	0.092	0.098	0.095	0.097	0.034	ID	0.967	0.965	0.976	0.975	0.973	0.956	0.955	0.956	0.876	0.877	0.875	0.876	0.907	0.909
34.KF679496. Australia. 1989	0.104	0.101	0.102	0.104	0.103	0.106	0.105	0.106	0.001	0.033	ID	0.996	0.986	0.984	0.983	0.98	0.978	0.979	0.879	0.88	0.877	0.879	0.906	0.908
35.KF679494. Australia. 1989	0.105	0.102	0.103	0.105	0.103	0.106	0.106	0.107	0.002	0.035	0.002	ID	0.983	0.982	0.981	0.978	0.977	0.977	0.878	0.879	0.877	0.879	0.905	0.907
36.KF679423. Australia. 1970	0.100	0.098	0.095	0.100	0.096	0.103	0.098	0.103	0.013	0.024	0.013	0.015	ID	0.996	0.994	0.969	0.968	0.969	0.877	0.878	0.875	0.877	0.906	0.908
37.AF058325. Australia. 1970	0.101	0.099	0.096	0.101	0.096	0.104	0.099	0.104	0.015	0.025	0.014	0.016	0.003	ID	0.994	0.969	0.967	0.968	0.877	0.878	0.875	0.877	0.905	0.907
38.KF679413. Australia. 1970	0.101	0.099	0.096	0.101	0.097	0.104	0.099	0.104	0.015	0.025	0.013	0.015	0.004	0.003	ID	0.967	0.966	0.967	0.876	0.877	0.875	0.876	0.903	0.905
39.KF679468. Australia. 2010	0.111	0.108	0.108	0.111	0.109	0.113	0.111	0.113	0.021	0.045	0.019	0.021	0.030	0.031	0.030	ID	0.996	0.996	0.876	0.877	0.875	0.877	0.897	0.899
40.KF679453. Australia. 2020	0.111	0.108	0.108	0.111	0.109	0.113	0.111	0.113	0.022	0.046	0.021	0.022	0.032	0.033	0.032	0.004	ID	0.996	0.873	0.875	0.873	0.874	0.896	0.898
41.KF679456. Australia. 2010	0.112	0.109	0.109	0.112	0.110	0.114	0.112	0.114	0.021	0.045	0.020	0.022	0.031	0.032	0.031	0.003	0.003	ID	0.874	0.875	0.873	0.875	0.895	0.899
42.MN026885. South Africa. 1969	0.140	0.144	0.141	0.146	0.140	0.145	0.142	0.147	0.137	0.138	0.135	0.136	0.138	0.138	0.137	0.140	0.143	0.142	ID	0.99	0.997	0.99	0.874	0.87
43.MN026897. South Africa. 2018	0.142	0.144	0.143	0.147	0.142	0.145	0.145	0.147	0.135	0.136	0.134	0.134	0.136	0.136	0.135	0.138	0.141	0.140	0.010	ID	0.99	0.986	0.872	0.869
44.MN026881. South Africa. 1969	0.141	0.144	0.142	0.147	0.141	0.146	0.143	0.147	0.139	0.140	0.137	0.138	0.140	0.140	0.138	0.141	0.144	0.143	0.003	0.010	ID	0.99	0.872	0.869
45.MN026880. South Africa. 1968	0.144	0.147	0.145	0.150	0.144	0.148	0.147	0.150	0.136	0.138	0.135	0.135	0.138	0.138	0.137	0.139	0.142	0.141	0.010	0.013	0.010	ID	0.871	0.868
46.MZ511168. Iran. 2020	0.078	0.083	0.086	0.086	0.087	0.087	0.089	0.089	0.098	0.098	0.098	0.099	0.100	0.100	0.101	0.110	0.111	0.112	0.137	0.139	0.139	0.141	ID	0.984
47.MZ511169. Iran. 2018	0.080	0.085	0.085	0.087	0.086	0.088	0.088	0.088	0.094	0.094	0.094	0.095	0.096	0.096	0.097	0.106	0.107	0.106	0.140	0.141	0.142	0.143	0.014	ID

The evolutionary distance analysis of Iranian strains showed similar results with identity. These strains had the highest and lowest evolutionary distances with the South African and the Middle East strains, respectively (Table 1).

### Phylogenetic analysis

**Fig. 1:**
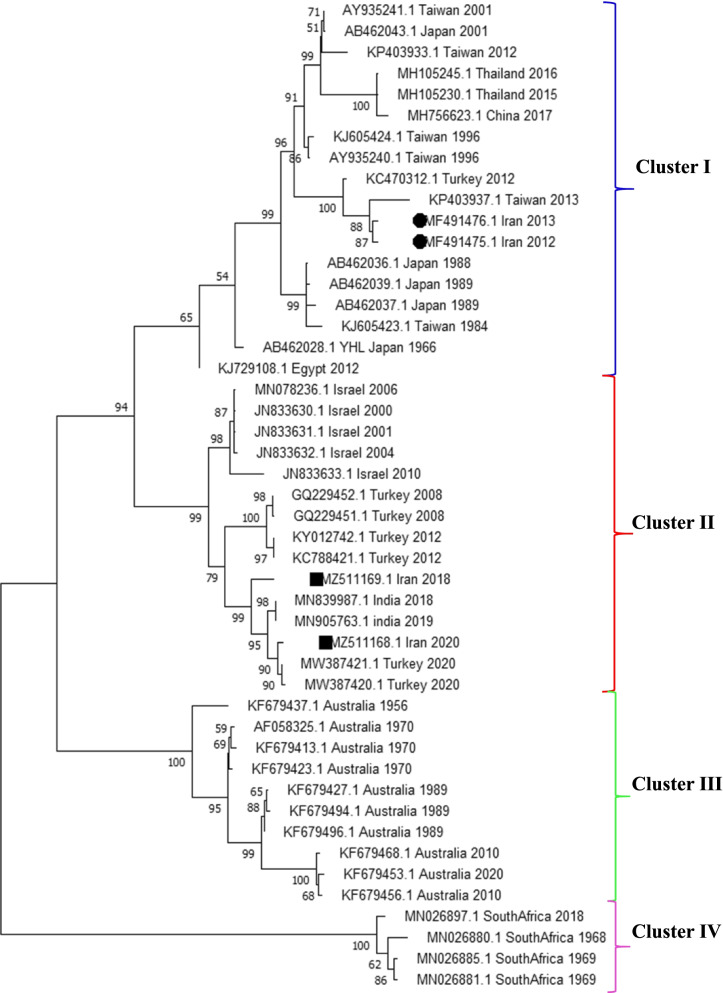
The phylogenetic tree of G gene ectodomain sequences constructed by the Maximum-likelihood using the MegaX program. BEFV strains were grouped into four clusters of I: East Asia, II: Middle East, III: Australia, and IV: South Africa. Sequencing viruses in this study are marked with a black square and previously studied viruses in Iran are marked with a black circle

The phylogenetic tree was constructed with the G ectodomain encoding sequences of the viruses. As shown in Fig. 1, the BEFV strains formed four major clusters of East Asian, Australian, South Africa, and Middle East strains. The IR-2018 and IR-2020 BEFV strains were grouped in the Middle East cluster with the Turkish, Indian, and Israeli strains. The viruses related to 2020 outbreaks in Turkey and Iran were placed on the same branch, while BEFV strains of Indian-2018 and 2019, Turkish-2020, and Iranian-2018 and 2020 formed a sub-cluster.

### Analysis of antigenic sites

**Fig. 2:**
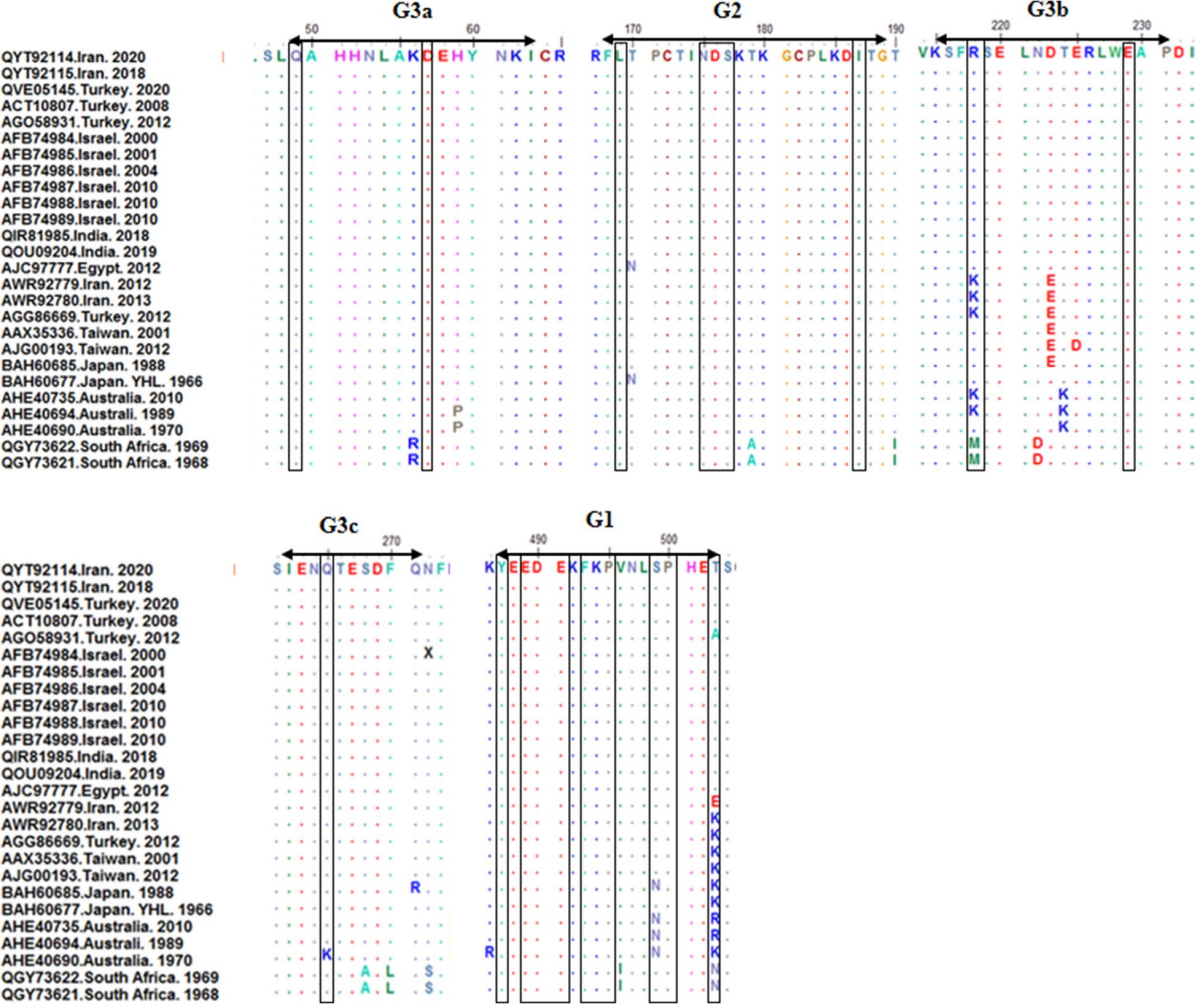
Alignment of deduced amino acid sequences of the neutralizing epitopes G1 (487–503), G2 (168–189), G3a (49–63), G3b (215–231) and G3c (262–271) on the G protein of different bovine ephemeral fever virus strains. Accession number, country and year of each strains were indicated on the left side, respectively. Putative glycosylation sites are boxed

No codon insertion, deletion, or frame shift was found in the glycoprotein sequences of IR-2018 and IR-2020 BEFV strains. All changes have resulted from single nucleotide substitutions. Multiple sequence alignments of G1, G2, and G3 antigenic sites showed that these neutralizing epitopes are highly conserved among the strains of the Middle East cluster, while it was found that the three substitutions at positions 218 (K to R), 223 (E to P), and 503 (K to T in IR-2013 strain and E to T in IR-2012 strain) in Iranian BEFV strains previously identified compared to present strains. Positions 218 and 503 are putative N-linked glycosylation sites. If the entire sequence of the G gene is considered, there are at least 22 amino acid substitutions between the strains previously identified in Iran and those present (Fig. 2). The Japanese YHL strain differed at 15 positions from IR-2018 and IR-2020 strains in which two substitutions of aa 170 (N to T) and aa 503 (K to T) were located in G2 and G1 antigenic sites, respectively.

## Discussion

Although BEF is one of the most important diseases of cattle in Iran [14], little is available about its agent and characteristics of circulating viruses. Therefore, in the present study, we characterized the prevalent BEFV strains in Khuzestan province southwest of Iran in 2018 and 2020.

In 2018, the incidence of BEF was sporadic and no report of the disease is available in other regions of the country. Our surveillance program of the BEF showed the absence of the disease in 2019. However, many animals suspected of contracting the three-day sickness were reported in Khuzestan province and other parts of the country in 2020. The RT-nested PCR results confirmed the occurrence of the disease in Khuzestan province as well as other regions of the country. The BEF disease epizootic has been reported in Turkey simultaneous with an outbreak in Iran [19]. The phylogenetic analysis, identity matrix, and distance evolution confirmed the high genetic closeness of prevalent strains in Turkey and Iran in 2020.

According to the phylogenetic analysis of the G gene ectodomain region, the BEFV strains were categorized into four clusters of East Asia, Australia, Middle East, and South Africa. However, previous studies have grouped the BEF strains into the three clusters of East Asia, Middle East, and Australia [20–24]. According to our results and the study of Omar et al [25], the BEFV strains of South Africa are distinct from those of other regions of the world and fall into the new cluster of South Africa. These strains showed the most distance evolution and the lowest similarity with the identified strains in the present study.

The phylogenetic tree revealed that the IR-2018 and IR-2020 strains are in the Middle East cluster close to Turkish, Indian, and Israeli BEFV strains. However, the viruses related to the outbreaks of 2012 and 2013 in Iran were grouped in East Asia cluster close to the strains from Taiwan, isolated in 2013, and Turkey, isolated in 2012. Most of the strains identified in Turkey belong to the Middle East group; however, in 2012, in some regions of Turkey, some identified BEF viruses were more similar to those placed in the East Asian group [16, 21, 26]. The BEF viruses that were identified in different regions of Iran during 2012 and 2013 were highly similar to those from Turkey [14]. Phylogenetic, identity matrix, and distance evolution analyzes revealed that the BEF viruses previously identified in Iran have a genetic distance from the strains of the present study and may have different sources. It seems that the virus circulating in 2012 and 2013 probably has expanded to the Middle East from East Asia [16, 26], while circulating strains of 2018 and 2020 were domestic strains of the Middle East. Studies of G gene nucleotide and deduced amino acid sequences from 2000 to 2020 indicate the circulation of a domestic strain in the Middle East. However, in 2012 and 2013, another strain that seemed to be exotic was prevalent in Turkey and Iran, but no evidence of the virus has been available since. It remains to be determined whether the structure of the G protein and its epitopes are effective in the compatibility of the virus with competence vectors [27].

This study demonstrated that BEFVs circulating in the Middle East are closely related phylogenetically and geographically. Pyasi et al. reported the outbreaks of BEFV during 2018 and 2019 in India [28]. Their molecular studies have shown that these strains were evolutionarily very close to those of Turkey and Israel, so they strongly suggested that these strains reached India from Israel and Turkey while maintaining their genomic sequence [28]. The findings of the present study also confirm this view. These viruses probably traversed across the countries like Turkey and Israel reaching Iran, and then India. Iran and India do not have a common border, so it is unclear exactly how the virus has been transmitted from Iran to India. The similarity between BEFVs from Iran’s and Turkey’s 2020-outbreaks (99.4–99.6% nucleotide similarity) was higher than that of IR-2018 and IR-2020 BEFVs (98.4% nucleotide similarity). The simultaneous outbreaks of BEFV in Iran and Turkey and high nucleotide and amino acid similarity indicate the common source of these viruses.

Comparison of G1, G2, and G3 antigenic sites showed that these neutralizing epitopes are highly conserved among the Middle East strains; however, the strains previously identified in Iran differed in three amino acids placed in G1 and G2 epitopes, which two of them were putative attachment site of oligosaccharides. Japanese YHL strain (vaccine strain used in Iran) differed from the Middle East BEFVs, especially IR-2018 and IR-2020 strains in G1 and G2 epitopes. The amino acid substituted in the G1 epitope is a putative N-linked glycosylation site; therefore, it may affect conformation and recognition of the neutralizing epitopes. Consistent with the differences in antigenic sites, Iranian and YHL BEFVs were placed as two distinct clusters. Due to these differences in antigenic structure, the use of heterologous strains as a vaccine may not provide full protective immunity [29]. According to the similarity of circulating strains in the Middle East, the use of these strains to develop vaccines can play an important role in preventing this disease.

## Conclusion

Comparison of the nucleotide and deduced amino acid sequences of the BEFV G gene related to different countries showed that these viruses are geographically divided into four clusters: Middle East, East Asia, South Africa, and Australia. Based on the chronology and geographical area, the outbreaks of Turkey (2020), Iran (2018 and 2020), and India (2018 and 2019) are proposed to be related, which also suggests that these strains can be used to develop a BEFV vaccine.

## Materials and methods

### Ethics approval

Written consent was obtained from all farm owners before entering the study. Sampling was performed by a specialist veterinarian to minimize pain and injury to the animal. In addition, the current study is approved by the Ethics Committee of Shahid Chamran University of Ahvaz. All methods were performed in accordance with the relevant guidelines.

### Collecting samples

This study is conducted in Khuzestan province, southwest of Iran, with a tropical climate. Heparinized whole blood samples were collected from cattle with clinical symptoms existing in affected farms. The most important clinical symptoms in these animals were persistent fever, muscle stiffness, constipation, ocular and nasal discharge, and spontaneous recovery after 3 days. A total of 50 and 40 samples were taken from suspicious animals in 2018 and 2020, respectively. Blood samples were centrifuged at 3000 rpm for 15 min to separate buffy coats. The buffy coat layer was transferred to a new tube and then rinsed three times.

### RNA extraction and RT-nested PCR

RNA was extracted from buffy coats using a viral RNA extraction kit (Bioneer; South Korea) according to the manufacturer’s protocol. Then, extracted RNA samples were reverse-transcribed using a kit (Yekta Tajhiz; Iran) to obtain cDNA. Screening of the positive BEFV samples was performed based on the amplification of the partial G gene via a nested PCR [30, 31]. Primer sequences are indicated in Table 2.

**Table 2:** List of primes used for detection and sequencing of glycoprotein (G) gene

Type		Primer Name	Sequence 5′-3′	Reference
Nested-PCR for Detection	First Run	G1F	ATGTTCAAGGTCCTCATAATTACC	32^1^
		G4R	AATGATCAAAGAACCTATCATCAC	
	Second Run	420F	AGAGCTTGGTGTGAATAC	31
		420R	CCAACCTACAACAGCAGATA	
Nested-PCR for sequencing	First Run	G1F	ATGTTCAAGGTCCTCATAATTACC	32^1^
		G4R	AATGATCAAAGAACCTATCATCAC	
	Second Run	G1F	ATGTTCAAGGTCCTCATAATTACC	32
		G1R	GCTTGTGTTGTATTAGGA	
		G2F	GGAATACGGAGATGAATCAA	32
		G2R	ATTCTGTTCTATCTGTGTGC	
		G3F	TTGAGGATGGAGAATGGTGG	32
		G3R	TACAACAGCAGATAAAAC	
		G4F	AAATGGAATGATCTTTGTGG	32
		G4R	AATGATCAAAGAACCTATCATCAC	

^1^G4R primer was modified based on the Middle East strains of India 2019 (MN905763) and Israel 2000 (JN833630)

### Sequencing the full-length G gene

BEFV G gene was amplified as described by Hsieh et al with some modification [32]. A nested PCR was designed according to primers introduced by Hsieh et al. In the first run, G1F and G4R primers were used to amplify an 1872 length fragment with the following protocol: 94 °C for 2 min, 25 cycles of 94 °C for 50 s, 50 °C for 50 s, 72 °C for 75 s, and a final extension of 72 °C for 5 min. In the second run, the PCR product was used as a template and G1-G4 fragments were amplified. PCR products were directly sequenced or subcloned into the pTG19-T vector (Vivantis; Malaysia) using standard techniques and then sequenced again. Sequencing was performed by Bioneer Company (South Korea) with the same PCR primers in two directions. Sequences were trimmed with BioEdit software version 7.0.4.1 (mbio, Inc., North Carolina, USA). Obtained sequences were submitted to the GenBank and are available under the accession numbers MZ51169 and MZ51168.

### Phylogenetic analysis

Available global nucleotide sequences of the BEFV G gene were retrieved from the NCBI Genbank database. All retrieved sequences from different countries and those of Iran generated in this study were aligned with ClustalW [33]. A phylogenetic tree was constructed based on G ectodomain encoding sequences using Molecular Evolutionary Genetics Analysis the MegaX software package (10.0.4) [34]. Tree construction was performed using Maximum-likelihood (ML) method, General Time Reversible (GTR) Model as the nucleotide substitution model [35], and gamma-distributed 4 (G4) according to a recent report [27]. Sub-tree Pruning & Re-grafting (SPR) branch swapping were applied to the analysis. The reliability of the branching was evaluated by the bootstrap analysis with 1000 replicates.

### Distance estimation and sequence identity matrix

The pairwise distance was estimated using MegaX software. The maximum composite likelihood was used as a substitution model. A discrete Gamma distribution (G) was used to model evolutionary rate differences among sites. The number of bootstrap replication was considered 1000. The sequence identity matrix was calculated using BioEdit software.

### Amino acid sequence analysis

Indicated nucleotide sequences of G encoding genes were translated to amino acid sequences using Expasy found at the expasy.org web server. Then, antigenic sites, including G1 (487aa–503aa), G2 (168aa–189aa), G3a (49–63), G3b (215aa–231aa), and G3c (262aa–271aa) [36] were retrieved and aligned with BioEdit software. These sequences were also compared with the Japanese Yamaguchi (YHL) vaccine strain, which is also used in Iran. In addition, the amino acid changes in putative N-linked glycosylation regions were also determined [18].

## Supplementary Information

**Additional file 1: Supplementary Table 1.** Amino acid identity of BEFVs from different countries according to glycoprotein.

## Data Availability

The datasets used and/or analyzed during the current study are available from the corresponding author on reasonable request. Sequence data of this project have been deposited in the GenBank of the National Center for Biotechnology Information (NCBI) under the accession number MZ51169 and MZ51168.

## Abbreviations

BEF: Bovine ephemeral fever
BEFV: BEF virus
ssRNA: single-stranded RNA
ML: Maximum-likelihood
GTR: General Time Reversible
G4: Gamma-distributed 4
SPR: Sub-tree Pruning & Re-grafting
